# Chronic complex dissociative disorders and borderline personality disorder: disorders of emotion dysregulation?

**DOI:** 10.1186/2051-6673-1-13

**Published:** 2014-10-14

**Authors:** Bethany L Brand, Ruth A Lanius

**Affiliations:** Towson University, Towson, MD 21252 USA; University of Western Ontario, London, ON N6A 5A5 Canada

## Abstract

Emotion dysregulation is a core feature of chronic complex dissociative disorders (DD), as it is for borderline personality disorder (BPD). Chronic complex DD include dissociative identity disorder (DID) and the most common form of dissociative disorder not otherwise specified (DDNOS, type 1), now known as Other Specified Dissociative Disorders (OSDD, type 1). BPD is a common comorbid disorder with DD, although preliminary research indicates the disorders have some distinguishing features as well as considerable overlap. This article focuses on the epidemiology, clinical presentation, psychological profile, treatment, and neurobiology of chronic complex DD with emphasis placed on the role of emotion dysregulation in each of these areas. Trauma experts conceptualize borderline symptoms as often being trauma based, as are chronic complex DD. We review the preliminary research that compares DD to BPD in the hopes that this will stimulate additional comparative research.

## Review

Dissociation is a process that provides protective psychological containment, detachment from, and even physical analgesia for overwhelming experiences, usually of a traumatic or stressful nature. Dissociation is conceptualized as analogous to the “animal defensive reaction” of freezing in the face of predation, when fight/flight is impossible [1, 2]. Dissociation is defined in the DSM-5 as a disruption of the usually integrated functions of: consciousness (e.g., trance states, non-epileptic seizures, pseudo-delirium); memory (e.g., impairment of autobiographical memory, that is, dissociative amnesia); awareness of body and/or self (depersonalization; e.g., feeling numb, watching oneself from a distance as if in a movie); environment (derealization; e.g., world appears far away or “foggy”; familiar places/people seem unfamiliar or strange; tunnel vision); and identity (e.g., confusion about one’s identity; experiencing discrete and discordant senses of self referred to as “identities”, which we refer to here as “dissociated self-states”; [3].

Trauma has been linked to difficulties with emotion regulation, particularly when it involves childhood abuse [4–6]. Studies of BPD patients have found that negative emotion, emotion dysregulation, and dissociation occurred simultaneously [7–9]. Researchers have found a connection between difficulties with emotion regulation and posttraumatic stress disorder [6, 10, 11]. Despite many researchers proposing that dissociation serves as a protection from overwhelming affect related to trauma [12–16], surprisingly few studies have empirically examined the relationship between trauma, dissociation, and emotion regulation. Briere [12] found that affect dysregulation and posttraumatic stress symptoms were the only significant multivariate predictors of dissociation in a traumatized sample. Briere concluded that risk factors including high posttraumatic stress and poor affect regulation skills may determine whether traumatized individuals develop pathological dissociation. He hypothesized that emotion regulation moderates the relationship between trauma and dissociation. However, a study of 290 college students found no evidence that emotion regulation moderated the relationship between peritraumatic dissociation or peritraumatic distress and later trait dissociation [17]. Rather, Kalill found that emotion regulation partially mediated the relationship between peritraumatic dissociation and later trait dissociation, but not between peritraumatic distress and later trait dissociation. Being less skilled at managing the trauma-related emotions appears to be a mechanism for dissociative symptoms developing or persisting after trauma.

### Overview of epidemiology, etiology, and clinical presentation

#### Etiology

One of the strongest predictors of dissociation is antecedent trauma, particularly early childhood trauma, as well as difficulties with attachment and parental unavailability [13, 18, 19]. The relationship between dissociation and many types of trauma is robust and has been validated across cultures in clinical and nonclinical samples using both cross-sectional and longitudinal methodologies as well as in large population studies and in prospective, longitudinal studies reviewed in [18]. In a meta-analysis of the trauma-dissociation relationship in 39 controlled studies, Dalenberg et al. found the average weighted effect size in *r* varied between .29 - .35 for various forms of childhood trauma. The trauma-dissociation relationship was even stronger in four studies comparing non-traumatized controls to individuals with dissociative disorders (*r* = .52 - .54).

Exposure to multiple types of trauma over multiple developmental epochs is associated with a wide range of clinical problems including emotion dysregulation (numbness, dissociation alternating with hyperarousal and emotional flooding); behavioral dysregulation (impulsive, self-destructive and aggressive behavior); identity problems including difficulties with body image and eating disorders; disruption in meaning (e.g., feeling life has no purpose); interpersonal problems; and somatization and medical problems including chronic fatigue, heart disease and autoimmune disorders [13, 20–23]. Many DD patients struggle with these difficulties e.g., [23]. Epidemiological studies have found that mood, somatoform, anxiety disorders, and substance abuse are commonly associated with antecedent trauma, as well as PTSD [24, 25]. These disorders are also common co-morbidities of patients with chronic complex DD e.g., [23, 26]. For example, in a prospective treatment study of DID and dissociative disorder not otherwise specified (DDNOS) patients, 89% also had PTSD (*n* = 242), 83% had a mood disorder (*n* = 226), 50% had an anxiety disorder other than PTSD (*n* = 136), 30% had an eating disorder (*n* = 81), 22% had a substance abuse/dependence disorder (*n* = 61), and 22% had a somatoform disorder (*n* = 59) [26].

There are many similarities between borderline personality disorder (BPD) and chronic complex DD, and DD and BPD have often been reported to occur comorbidly [27–32]. Individuals with DDs and BPD also frequently experience major fluctuations in identity and emotional states, and depersonalization and derealization during stress, as well as exhibit high rates of self-harm and suicidality e.g., [33–36]. Suicidal and/or self-injurious behaviors are common among DD patients with 67% of DD patients reporting a history of repeated suicide attempts and 42% reporting a history of self-harm [33, 34, 37]. The majority of BPD patients (60%-70%) also make suicide attempts [38]. A recent study has noted that patients diagnosed with both BPD and DID were more symptomatic as compared to individuals who were diagnosed with either diagnosis alone [31].

Trauma experts conceptualize borderline symptoms and DD as being based in trauma and attachment difficulties, which contribute to difficulties with affect regulation e.g., [13, 15, 22, 39–41]. Individuals with DID report rates of childhood sexual abuse (CSA), childhood physical abuse or both with 95 - 97% frequency e.g., [34, 42] compared to somewhat lower ranges between 80-96% for individuals with borderline personality disorder BPD; [40, 43–45]. A study of 214 BPD outpatients found an overall childhood abuse rate of 81% with only 44% reporting CSA [43]. Lifetime rates of trauma in BPD tend to be high. For example, in a recent multi-center study of 135 BPD patients, 96% reported lifetime trauma, 48% of the women and 28% of the men reported sexual violence, and 65% reported severe physical violence [44]. The age of onset may be earlier and abuse may be more severe and chronic in DD compared to BPD, although research needs to be conducted to further examine this hypothesis.

The overlap in symptoms partly contributes to the high comorbidity frequently observed between BPD and DD. BPD is diagnosed in 30% to 70% of DID patients [32, 46–51]. DD are diagnosed in 41% to 72% of BPD patients [28, 29, 32, 44],]. Personality disordered patients who have dissociative symptoms and identity disturbances may be misdiagnosed as DID. For example, in the cluster of symptoms in “impairment in identity”, both “unstable self-image” and “dissociative states under stress” are DSM-5 criterion symptoms for borderline personality disorder. Individuals with both types of disorders report dissociative amnesia. However, college students with BPD reported greater awareness of dissociative amnesia than did the DD group; the students who had both BPD and a DD reported the highest level of amnesia [52].

Studies of BPD patients also find high rates of trauma disorders. For example, 79% of a BPD sample had PTSD, 55% had complex PTSD, and 41% had DD in one recent study [44]. DD experts theorize that severe, dysregulated PTSD and dissociative symptoms account for global instability that leads to the high rate of BPD diagnosis, with only a minority of DID patients meeting full BPD criteria after stabilization of acute symptoms [53, 54]. Longitudinal studies are needed that compare the rates, severity, and age of onset across the range of childhood and adult traumas, and the range of trauma disorders (PTSD, complex PTSD, BPD, and DD) before and after initial stabilization of trauma-related symptoms for patients with BPD and DD.

#### Epidemiology

Epidemiological studies of DD have been conducted in the United States, Canada, the Netherlands, Germany, Switzerland, Finland, China, and Turkey. The most common type of DDNOS, which has been replaced in the Diagnostic and Statistical Manual of Mental Disorders-5, called other specified dissociative disorder (OSDD), is typically found to be the most prevalent DD in general population and clinical studies with a prevalence rates up to 8.3% in the community reviewed in [23]. Across general population studies, the most severe DD, dissociative identity disorder (DID) has a prevalence of approximately 1% and has been found in .4 – 14% of psychiatric inpatients and outpatients, depending on the sample [55]. The lifetime prevalence of BPD has been estimated to be 5.9% [56], and BPD is thought to be present in 7 – 27% of psychiatric outpatients and contribute to 3.8 lifetime psychiatric hospitalizations [57].

Most DDNOS/OSDD patients are similar in presenting symptoms, history, clinical course, and treatment response to DID patients, so DDNOS/OSDD is combined with DID here (reviewed in [23]). DID is conceptualized as a childhood onset, posttraumatic developmental disorder in which the child is unable to consolidate a unified sense of self due to severe, chronic childhood abuse, often involving a caretaker [13]. Dissociation during and after the repeated episodes of abuse allows the child to psychologically detach from the emotional and physical pain, in turn potentially resulting in alterations in memory encoding and retrieval [58]. Over time, this leads to fragmentation and compartmentalization of memory, as well as difficulty retrieving memory [13, 23, 59]. Exposure to early, typically chronic, trauma results in the elaboration of discrete physiological, psychological, and behavioral states that can persist and, over later development, become increasingly developed, ultimately resulting in dissociative emotional/behavioral/memory self-states [13].

#### Clinical presentation and comorbidity

Many clinicians and lay people believe that DID presents with dramatic, florid personality states with obvious state transitions (switching). These florid presentations are likely based on media stereotypes, but actually occur in only about 5% of DID patients [60]. The vast majority of DID patients have subtle presentations characterized by a mixture of dissociative and PTSD symptoms embedded with other symptoms such as posttraumatic depression, substance abuse, somatoform symptoms, eating disorders, personality disorders, and self-destructive and impulsive behaviors [23, 61]. A classic presentation includes a history of multiple treatment providers, numerous serious suicide attempts resulting in repeated hospitalizations, and good medication trials typically with limited or no benefit [23].

Although the media and public are often overly fascinated with DID dissociated self-states, the complex symptomatic presentation of DID receives more clinical attention from trained clinicians [62–64]. Under-recognition of DID is common because the most obvious and pressing aspect of a patient’s clinical presentation may be one of the many comorbid disorders (e.g., severe mood disorders, posttraumatic stress disorder [PTSD], eating disorder, substance abuse, BPD), or the pseudopsychotic symptoms related to the overlap and intrusions of self-states into consciousness. This overlapping influence of self-states causes “passive influence” phenomena or Schneiderian first rank symptoms, which are more common in DID than overt, obvious “switching” of states. DID patients experience more first rank symptoms than do individuals with schizophrenia, with the exception of thought broadcasting or audible thoughts [65, 66]. Intrusions into consciousness may be partially excluded from consciousness (e.g., hearing voices of states, thought insertion/withdrawal, “made” actions/impulses) or fully excluded from consciousness (e.g., time loss, fugues, disremembered behaviors; [61, 62, 64]. Psychotic symptoms occur in 20 – 50% of BPD patients and childhood trauma may play a role in development of hallucinations in BPD, as well as in DD and other disorders [67]. Research indicates that DD patients show greater ability to be logical, reflective, and reality-based than BPD patients (see below) although more research directly comparing the groups on psychotic symptoms, including Schneiderian symptoms, are needed.

### Neurobiology of dissociation: common underlying neural pathways in PTSD, BPD, and chronic complex DDs

Two subtypes of acute trauma response have been found in a range of neurobiological studies, one primarily involving dissociative symptoms and the other predominantly intrusive, hyperaroused symptoms; these symptoms may underlie some forms of emotion dysregulation in trauma-related disorders, including PTSD, chronic complex DDs, and BPD [68–70]. These two subtypes of dissociation have been described as primary and secondary dissociation [16]. Primary dissociation refers to the re-experiencing/hyperaroused type of dissociation and includes the classic PTSD symptoms including intrusive recall, flashbacks, and nightmares. In contrast, secondary dissociation is characterized by such symptoms as numbness, depersonalization, derealization, and analgesia responses [16].

The neuronal circuitry underlying re-experiencing/hyperarousal (primary dissociation) and depersonalization/derealisation dissociative (secondary dissociation) responses in PTSD predominantly related to childhood abuse has been studied by Lanius and colleagues [71–76] using the script-driven, symptom-provocation paradigm. Patients’ sensory-rich trauma narratives were read to them while they recalled the traumatic memory vividly during an fMRI scan. Approximately 70% of patients relived their traumas showing primarily a re-experiencing/hyperarousal response with an increase in heart rate [75]; in contrast, 30% showed a secondary dissociative response with no concomitant increase in heart rate [73, 74]. In comparison to controls, patients who had a hyperaroused, reliving response showed significantly less activation of the thalamus, anterior cingulate gyrus (BA 32) and medial frontal gyrus (BA 10,11), occipital lobe (BA 19) and inferior frontal gyrus (BA 47) [75]. Less activation of the anterior cingulate and medial prefrontal were consistent with PET script-driven imagery studies of sexual abuse and combat-related PTSD e.g., [77–81]. Striking differences occurred in patients who exhibited secondary dissociation while hearing their trauma scripts [74]; they showed *higher* brain activation in the superior and middle temporal gyri (BA 38), the medial prefrontal cortex (BA 9), the anterior cingulate gyrus (BA 24 and BA 32), the inferior frontal gyrus (BA 47), the occipital lobe (BA 19), and the parietal lobe (BA 7). The neural correlates of hyperarousal vs. hypoarousal in patients with PTSD showed the *opposite* patterns of activation in brain regions that are implicated in arousal modulation and emotion regulation. In particular these differential patterns are found in the medial prefrontal cortex and the limbic system, including the anterior cingulate cortex and the amygdala.

#### Failure of corticolimbic inhibition

Along with impaired cortical modulation, research shows increased activation of the limbic system, especially the amygdala, which is involved in fear conditioning, among PTSD patients [82–84]. Studies in PTSD patients have also reported direct inhibitory influence of the prefrontal cortex on the emotional limbic system [81, 85]. Thus, the *low* activation of medial prefrontal regions described in the re-experiencing/hyperaroused PTSD subgroup is consistent with failed inhibition of limbic reactivity and is associated with re-experiencing/hyperaroused emotional undermodulation. These symptoms are a form of emotion dysregulation that involves emotional undermodulation, mediated by failure of prefrontal inhibition of limbic regions. Furthermore, the severity of re-experiencing was *positively* correlated with activation in the right anterior insula (a region involved in somatic aspects of emotional states and interoception of emotions), and *negatively* correlated with activation of the rostral portion of the anterior cingulate cortex (a region involved in arousal and emotion regulation). These findings are consistent with the phenomenology and clinical presentations of PTSD patients who exhibit pathological emotional undermodulation during re-experiencing/hyperarousal flashback states, including a variety of negative emotional states such as irritability and anger and associated bodily experiences.

Failed corticolimbic inhibition has also been observed in individuals with BPD and has been suggested to underlie symptoms of emotion dysregulation, i.e. hypersensitivity and hyperresponsivity to emotional stimuli often observed in this disorder reviewed by [86–88], and resting state functional network connectivity has been shown to involve decreased connectivity of emotional regulatory networks in BPD [89]. Heightened limbic reactivity particularly affecting the amygdala and insula has been a well replicated finding in response to exposure to negative emotional stimuli in BPD [90–96]. Moreover, hyporeactivity of frontal regions has frequently been observed in response to emotionally arousing stimuli in BPD, including emotional faces and pictures, trauma-related stimuli) [97–101] in addition to cognitive reappraisal of negative emotional stimuli [92, 96]. Altered brain activation in the orbitofrontal cortex has also been suggested to be associated with symptoms of impulsivity in BPD [102].

#### Excessive corticolimbic inhibition

In contrast to the re-experiencing/hyperaroused group, the secondary dissociative group showing symptoms of depersonalization and derealization during traumatic memory recall exhibited abnormally *high* activation in the anterior cingulate cortex and the medial prefrontal cortex [74]. The dissociative PTSD patients showed an emotional overmodulation in response to exposure to traumatic memory recall mediated by midline prefrontal inhibition of the limbic regions. The dissociative response was *negatively* correlated with right anterior insula activation and *positively* correlated with activation in the medial prefrontal cortex and dorsal anterior cingulate cortex [71]. The medial prefrontal cortex that was *positively* correlated with hyperaroused symptoms was *negatively* correlated with amygdala activity during script-driven imagery for the hypoaroused dissociative group [103]. These results provided the neurobiological basis of the dissociative subtype of PTSD which focuses on symptoms of depersonalization and derealization.

Evidence for excessive corticolimbic inhibition during secondary dissociative states has also been described in BPD during pain and working memory processing as well as at rest. Studies examining pain sensitivity after script-induced state dissociation in BPD comorbid with PTSD have reported lower pain sensitivity and higher brain activation of the left cingulate cortex [104]. During an adapted Sternberg working memory task, patients with BPD exhibited a negative correlation between limbic brain regions, including the amygdala, and self-reported symptoms of secondary dissociation [93]. Similarly, in PTSD, a negative correlation between state secondary dissociation and amygdala activation has been reported during pain processing [105]. Moreover, preliminary results point to increased resting state functional connectivity between the left amygdala and right dorsolateral prefrontal cortex as a function of increased trait dissociation (DES scores) in BPD [106]. These findings may suggest increased top down regulation of the amygdala at rest with increasing levels of trait dissociation in individuals with borderline personality disorder.

Excessive corticolimbic inhibition was first proposed by Sierra and Berios in the context of depersonalization disorder and was thought to underlie dampened emotional responses in this disorder [14]. Previous neuroimaging studies in DPD support this model in that they have demonstrated diminished subcortical limbic activity in response to increasingly intense happy and sad face stimuli [107]. Moreover, negative correlations were observed between psychophysiological measures and skin conductance response in patients with DPD but not in healthy controls. An additional study demonstrated increased activation of the right anterior cingulate and medial prefrontal cortex during self vs stranger face processing; activation in the medial prefrontal cortex positively correlated with depersonalization symptoms [108]. Interestingly, a recent study showed that a single session of rTMS administered to the right ventrolateral prefrontal cortex significantly reduced symptoms of depersonalization and enhanced psychophysiological arousal in DPD patients [109] also see [110], thus suggesting that such treatment may be one way of altering top down regulation of limbic regions.

In patients with DID, two different identity states that have differential access to the traumatic memories have been examined: a neutral state that generally cannot access the traumatic memories and is thus not overwhelmed by the memories and a traumatic state that has full access to the memories and often re-experiences them in the form of flashbacks [111]. Differential brain activation patterns among the two states have been observed. The traumatic state shows brain activation patterns more consistent with re-exeriencing/hyperarousal states in PTSD whereas the neutral state exhibits neural responses more consistent with excessive cortocolimbic inhibition, including decreased autonomic reactivity. Differential brain activation in these two states has also been observed during neutral and angry face processing [112]. A resting state SPECT study in DID has shown increased activation of superior and middle frontal regions as well as decreased perfusion of orbitofrontal as compared to controls [113]. During virtual maze performance, patients with DDs showed less activation of the cingulate cortex, insula, and inferior parietal cortex as compared to controls, and the performance of DD patients improved as a function of increasing dissociative disorder severity [114]. Future studies will need to clarify the potential role of corticolimbic inhibition during different neuroimaging paradigms.

In summary, the neurobiological model of excessive limbic inhibition underlying secondary dissociative symptoms, including symptoms of depersonalization and derealization appears to be supported in number of trauma-related disorders, including PTSD, DID, and BPD. Future research examining the neurobiological and psychophysiological effects of dissociative symptoms in a transdiagnostic fashion [115, 116] are therefore warranted. Moreover, expanding this model to include other pertinent brain regions involved in secondary dissociative responses will be a priority.

### Psychological profiles of chronic complex DD and BPD Patients

Few studies have directly compared the clinical profiles of BPD to DD samples using psychometric instruments, although a few studies have compared data from DD samples to published BPD norms. Even research that examines just BPD or DD using standardized personality tests such as the MMPI-2 is relatively sparse. Some studies utilizing self-report personality tests indicate areas of similarity between BPD and DD patients, although studies using the Rorschach have found important personality differences.

#### Millon Clinical Multiaxial Inventory (MCMI-II)

DID patients generally have similar MCMI [117] profiles compared to BPD individuals, although DID patients were 200 base points higher than MCMI’s BPD norms on schizoid, avoidant and schizotypal scales see [30, 118]. DD patients receive high scores on MCMI scales for Avoidant, Dependent, Passive-aggressive, and Borderline personality disorders [30, 118]. No studies have directly compared individuals with BPD and DD using the MCMI-II.

#### Minnesota Multiphasic Personality Inventory (MMPI/MMPI-2)

In a study of 53 DID patients’ MMPI-2 [119] profiles, the highest elevation occurred on scale Schizophrenia (8) with additional marked elevations (>70 T) on scales Depression (2), Paranoia (4), Psychopathic Deviate (6), and Psychasthenia (7) which measures anxiety [120]. The DID Schizophrenia-Depression- code type has been found in previous DID samples [121], although Schizophrenia combined with Paranoia or Psychopathic Deviate (due to family discord and self-alienation, rather than problems with authority) are also common [122–125]. Elevations in Schizophrenia among interpersonally traumatized individuals are frequent [126–129]. In fact, content analysis reveals that several items on this scale are cardinal symptoms of dissociation (e.g., daydreaming; feeling numb; gaps in memory; feeling unreal), so it is not surprising that the Schizophrenia scale is correlated with severity of dissociation among DID patients r = .47, [120]. BPD patients have elevations on Schizophrenia, Paranoia, and Psychasthenia with additional elevations on Psychopathic Deviate, Psychasthenia, and Depression [130]; BPD scores tend to be somewhat less elevated than those found in DD patients, although no study has directly compared individuals with both disorders.

#### Personality Assessment Inventory (PAI)

A sample of 78 BPD patients (2/3 of whom were hospitalized) were described in the PAI [131] manual as having scored highest on Suicidal Ideation and Borderline Features subscales, followed by Depression and Anxiety [131]. In comparison, in the only PAI study of DD patients, forty-two DID/DDNOS inpatients scored highest on Suicidal Ideation and Depression, followed by Anxiety Related Disorders and Schizophrenia [132]. The DD patients were also in the clinically impaired range on Traumatic Stress, Anxiety, and Borderline features subscales (mean T score = 73.87, standard deviation = 10.26). In fact, the DD group scored in the same range as the BPD group on all four of the Borderline subscales. In contrast to being portrayed in movies as antisocial and violent towards others, DD patients are typically low on aggression, antisocial features and mania, as well as being fearfully avoidant of others e.g. [30, 132].

#### Rorschach

The Rorschach Comprehensive System RCS; [133] has documented distinctive personality traits of DD patients [134–137]. Two studies compared DD patients’ protocols to published BPD norms [138] to determine which features distinguished the disorders [134, 137]. DID patients demonstrate a heightened capacity for introspection (FD) and cognitive complexity (blends) when compared with Exner’s norms for BPD patients [134]. A sample of DID patients showed a tendency to become caught up in inner experience (lower Lambda scores) and significantly higher capacity for intellectual self-reflection (high FD and V scores) when compared to BPD patients [137]. Scroppo et al. suggested that a fundamental difference between DID and BPD was the tendency among dissociative individuals to “elaborate upon and imaginatively alter their experience” (p. 281) in contrast to BPD patients, who simplify experience and respond in an affectively driven manner. This lead Scroppo et al. to the conclusion that DID is a relatively distinct diagnostic entity from BPD, one that is more “imaginatively based” and relies upon a “cognitively complex response style”.

Brand and colleagues replicated and extended these findings in their comparison of 67 DID patients to 40 BPD patients [136]. Compared to the BPD group, the DID group showed greater: self-reflective capacity (higher FD), ability to modulate emotion (lower CF + C – FC), social interest (higher Sum H), accurate perception and logical thinking (lower X- and WSUM6), and ability to see others as potentially collaborative (higher COP). Individuals with DID also produced an unusual degree of introspection (FD = 2.72), suggestive of “self-consciousness and soul-searching” [139], p. 160. The large between-group effect size in FD suggests that this trait may helpful in differential diagnosis. The greater capacity for or tendency toward introspection in individuals with DID versus BPD may allow DID patients to reflect on their behaviors and feelings in relationships, including the therapeutic relationship, possibly enabling them to benefit more easily from insight-oriented therapy.

A study comparing four treatment seeking groups (+BPD + CSA, +BPD –CSA, -BPD + CSA, -BPD –CSA) found that the clinician-rated severity of CSA predicted affect dysregulation as well as difficulties with reality testing on the Rorschach [140]. Future research should compare the severity of CSA, as well as other types of trauma, in predicting affect dysregulation in BPD and DD patients.

Although DD are associated with tremendous suffering including the loss of a continuous sense of one’s self and one’s memory, theorists suggest that dissociation provides some protection from the overwhelming danger and tumultuous emotions [13, 141]. Perhaps BPD patients could not find as ready an escape as could DD patients from early adverse experiences. Armstrong [141] theorized that their greater ability to dissociate places DD individuals on an atypical developmental pathway, rather than leading to developmental arrests that are theorized to occur with BPD individuals [142]. Due to having periods of amnesia for some of the trauma they experienced, often perpetrated by the caregivers they loved and were dependent upon e.g.; [53], DD individuals may have been able to, despite being maltreated, continue to view others as potentially caring, at least in some self-states. Their increased ability to compartmentalize and "not fully know," thereby partly avoiding feelings of betrayal and awareness of the aggression of others, paired with other personality traits documented earlier, may contribute to DD patients’ participation in at least some supportive relationships. Findings of personality strengths, especially in relational capacity, are in line with research showing that dissociation preserves attachment even in the face of betrayal and abuse by caregivers [58].

In summary, across psychological tests, DD patients report a range of psychological difficulties that likely developed as a result of severe childhood abuse and disturbances in attachment. Studies indicate DD patients have difficulties with affect regulation, which they attempt to regulate through self-harm, suicidal thoughts and behaviors, and dissociation. Despite exhibiting elevations on self-report measures of schizophrenia, their thinking appears to be more logical, reflective, and reality-based than that of many BPD patients. The psychological profiles of DD patients are consistent with neurobiological findings: DD patients show difficulties with emotion regulation, with a resultant tendency to vacillate between hypoarousal and dissociative states, and emotional flooding including profound depression and intense anxiety.

### Treatment implications

As a transdiagnostic feature, dissociation is crucial to assess because it has often been shown to have a negative impact on treatment outcome, even for a variety of non-DD psychiatric disorders including BPD e.g., [143–153] but also see [144, 154]. Psychotherapy has been reported to be more difficult in individuals with DID who have comorbid BPD [155, 156]. Dissociation inhibits amygdala-based emotional learning in patients with BPD [157] and may thus interfere with the psychotherapeutic process. In support of this hypothesis, dissociation has been shown to be associated with response to treatment among acutely traumatized individuals. Specifically, dissociation *during treatment sessions* was the only significant predictor among several variables in predicting poor response to early intervention for individuals treated at an emergency department [158]. Individuals with PTSD and mild to moderate dissociation have shown different responses to trauma treatments [159, 160]. Moderately dissociative patients responded better to phase-oriented treatment that provided emotion regulation skills training prior to doing trauma exposure narrative work, as compared to doing exposure without skills training [159]. A trial of standard exposure therapy found a similar rate of improvement among low and moderate dissociation groups, although 10% of the low dissociation versus 69% of the moderate dissociation group met PTSD criteria at follow-up, suggesting that those with moderate levels of dissociation will need longer treatment to achieve good outcomes [144]. A study of refugees treated with exposure therapy found reductions in PTSD symptoms regardless of level of dissociation, as measured by the two dissociation items on the Clinician-administered PTSD Scale CAPS; [154, 161]. Despite improvements overall, those with severe depersonalization did not show as large a decrease in depression as did those with low depersonalization (*g* effect size of .35 versus .80, respectively). It is important to note that studies such as the one by Halvorsen and colleagues that rely on two CAPS items to assess dissociation may miss the range and severity of dissociative symptoms, given that dissociation is recognized as a multidimensional construct [162]. In a study of exposure therapy conducted with adults who had experienced complex trauma, 45% of whom were in the range of dissociation suggestive of a DD, patients showed a worsening of symptoms, including a trend level worsening of a physiological marker of emotion regulation (respiratory sinus arrythmia; [163]). Despite exposure therapy being considered a first-line treatment for PTSD [164], this severely dissociative sample did not benefit from exposure therapy; rather, they showed more improvement in response to psychodynamic treatment and stress inoculation therapy. The participants in the Hagenaars et al. study had comparably less severe levels of dissociation and less severe traumas than those in D’Andrea & Pole’s study (e.g., 24% vs. 67% sexual assault). Given that state dissociation has been shown to interfere with emotional learning [157] and that dissociation during treatment sessions has been shown to be the only predictor of treatment outcome, it is crucial that future studies examine the effects of state dissociation *during treatment sessions* on treatment outcome rather than only examining the effects of trait dissociation on psychotherapeutic processes in general, as most studies have done to date. It will also be critical that future studies utilize measures of dissociation that fully assess the range and severity of dissociative phenomena.

The differences in severity of dissociation and trauma across treatment samples likely also contribute to differences in outcomes observed. In support of this possibility, several studies have found that traumatized patients with the highest level of dissociation do not respond as well to treatment as those with lower levels of dissociation [160, 165, 166] but see [154]. However, even very high levels of dissociation did not preclude improvement in a range of outcomes including comprehensively assessed dissociation in DD patients receiving complex trauma inpatient treatment. However, despite improvements in depression and general psychiatric distress, DD patients did not show as much improvement in dissociation, PTSD or interpersonal problems when compared to non-DD traumatized patients in treatment that did not focus on identity fragmentation and amnesia [166]. At discharge, only 4.3% of the DD group showed reliable change in dissociation compared to 24.2% of the non-DD group in a treatment that lacked specific focus on treatment of amnesia and dissociative self-states.

Most of the current research suggests that treatment for complex trauma individuals with moderate and severe dissociation should be staged such that emotion regulation skills and management of dissociative symptoms are taught before trauma processing begins. The staged approach is advocated by experts in treating DD patients [167, 168], as well as expert recommendations for treating complex trauma [169, 170]. Dialectical behavior therapy (DBT) is a stage-oriented treatment approach that has been used in the treatment of BPD with and without comorbid PTSD; when DBT was followed by prolonged exposure therapy, it was found to reduce PTSD, dissociation, shame, depression, suicide attempts and self-injury [171]. This study used DBT to improve emotion regulation, mindfulness and interpersonal effectiveness skills, thereby addressing dissociative symptomatology, during the first phase of treatment prior to using exposure. Other studies have found skills based training followed by trauma processing to be an effective treatment for BPD with PTSD [172, 173].

DD patients are often extraordinarily fearful of inner and external reminders of trauma and the related emotions that such reminders trigger e.g., [174]. According to the structural model of dissociation, there is a core phobia in DD individuals who avoid the full realization of trauma and its effects. Steele and colleagues conceptualized the staged treatment of DD as overcoming various phobias, including of dissociated self-states, emotions, attachment, traumatic material, and intimacy [174]. This phobia of trauma and emotion, often structuralized into dissociated self-states that often vary by emotional and traumatic content, must gradually be understood and resolved during therapy, along with overcoming fundamental skill deficits in affect regulation, among other treatment tasks.

## Conclusions and future research

Emotion dysregulation may be a mechanism linking trauma to dissociation. It is also an important feature of DD and BPD. Epidemiological, assessment, neurobiological, and treatment outcome research needs to focus on emotion dysregulation in disorders with dissociative symptomatology including DD and BPD. To enhance our understanding of the similarities and differences between DD and BPD, and to lead to research that can clarify causal mechanisms and yield more targeted treatments, future research needs to study the range of trauma exposure as well as the range of dissociation, particularly dissociation that occurs during treatment sessions, using clinical and community samples. This knowledge will help to develop interventions that are most effective for the treatment of dissociation as well as improve our understanding of how specific interventions, such as improving affect regulation, can be employed for patients with a range of dissociative psychopathology.
